# The Stiff People: Two Rare Cases of Stiff-person Syndrome

**DOI:** 10.7759/cureus.1602

**Published:** 2017-08-23

**Authors:** Muhammad Z Siddiqui, Hassaan Tohid, Rebecca Brown, Samia Qazi

**Affiliations:** 1 Internal Medicine, Nassau University Medical Center, East Meadow, USA; 2 Research, California Instititute of Behavioral Neurosciences and Psychology, Fairfield, USA; 3 Neurology, New York Institute of Technology College of Osteopathic Medicine, Long Island, USA; 4 Internal Medicine, New York Institute of Technology College of Osteopathic Medicine, Long Island, USA

**Keywords:** stiff-person syndrome, sps, gamma butyric amino acid, gaba, glutamic acid decarboxylase, gad, stiff-man syndrome

## Abstract

Stiff-person syndrome (SPS) is a rare disorder that affects the central nervous system and is characterized by progressive muscle stiffness, rigidity, and spasm of axial and limb muscles. The syndrome is caused by a lack of gamma aminobutyric acid (GABA), which occurs because of antibodies against glutamic acid decarboxylase (GAD), an essential enzyme for GABA synthesis. Hence, the patients present with increased muscular activity. In this article, we will discuss two case studies of stiff-person syndrome.

## Introduction

Distinct from the muscular rigidity of Parkinson’s and muscular spasms of tetanus, stiff-person syndrome (SPS) is a rare disorder that affects the central nervous system. It is characterized by progressive muscle stiffness, rigidity, and spasm of the axial and limb muscles [1]. A proposed mechanism of SPS is the presence of antibodies against glutamic acid decarboxylase (GAD), an essential enzyme in the synthesis of γ-aminobutyric acid (GABA). This results in decreased inhibition of the central nervous system, leading to increased muscle activity. Herein, we present one case of partial stiff-person syndrome presenting as lower limb rigidity and another case of generalized stiff-person syndrome.

## Case presentation

Case 1

An 84-year-old Caucasian male presented to the emergency department after a fall in his home. On presentation, the patient was alert and awake but disoriented. Mild swelling was observed on the right side of the head. The patient was severely diaphoretic, tachycardic with systolic hypertension, and had muscular spasticity in the lower extremities bilaterally. The patient was unable to move his legs or ambulate. He denied any loss of consciousness, confusion, seizure, numbness, weakness, or sensory loss. Vitally, the patient had a pulse of 105, blood pressure of 154/56, respiratory rate of 18, and body mass index (BMI) of 22.8.

A computed tomography (CT) scan of the head and cervical spine showed no acute intracranial pathology, acute vertebral compression fracture, or gross subluxation. No acute vertebral compression fracture was seen on the X-ray of the pelvis. Past medical history was significant for hypertension, hyperlipidemia, and prostate cancer with seed implants. The patient was a social drinker with no history of smoking. His family history was remarkable for diabetes mellitus type 2 in his mother.

On further evaluation, it was found that the patient had been treated for episodes of severe rigidity in his back and lower extremities bilaterally in a nearby hospital. The records were obtained and they revealed that he was positive for anti-GAD 65 antibody. The patient was diagnosed with SPS and intravenous immunoglobulin (IVIG) was administered, which as per records showed improvement in the patients' symptoms.

The patient was initially managed with gabapentin and clonazepam, resulting in decreased spasticity. Later, the patient was started on IVIG with minimal improvement in his symptoms. Episodes of spasms were observed, lasting up to three hours and the rigidity was relieved by IV lorazepam. Although lorazepam and IVIG therapy have been helpful in improving acute symptoms, the patient has continued to experience episodic muscle spasms with increasing frequency and severity for a duration of three years.

Case 2

A 26-year-old female presented to the emergency department with a history of immune deficiency since birth. Since the age of 11, she concurrently exhibited symptoms of neuropathy, Bell’s palsy, and muscle spasms. These symptoms progressed to worsening dystonia and gait disturbance, rendering her unable to ambulate by age 16. Later on, she was diagnosed with common variable immune deficiency (CVID) at the age of 18. Furthermore, at age 24, a diagnosis of a rare neurological condition known as stiff-person syndrome (SPS) was made. Her SPS symptoms improved greatly after initiation of low doses of intravenous immunoglobulin (IVIG) twice monthly for the CVID. She slowly regained the ability to walk, effectively treating both rare conditions with one therapy.

## Discussion

With an incidence of about one per million per year, stiff-person syndrome is a rare disorder recognized by its characteristic episodes of fluctuating muscular spasms and hypertonicity. Although classic stiff-person syndrome includes muscle rigidity throughout the entire body, the axial musculature as well as the musculature of the limbs are almost always involved [2-3]. A variant of classic stiff-person syndrome is partial stiff-person syndrome, where the muscular spasms are limited to either the upper or lower limbs, and the trunk is spared [4]. In our first case, the patient suffered a partial stiff-person syndrome in which he experienced episodic, spasmodic muscular rigidity and pain in his lower limbs. The patient in case 2 suffered from generalized stiff-person syndrome symptoms during attacks.

The presence of anti-GAD antibodies is theorized to be the cause of stiff-person syndrome via the inhibition of glutamic acid decarboxylase (GAD), which is a critical enzyme in the inhibitory pathway of the central nervous system (CNS). Due to the decreased levels of GABA, which results from the inhibition of GAD, muscle activity is uninhibited and subsequently increases. This is thought to be the mechanism behind the typical muscular spasm and rigidity of stiff-person syndrome, with specific anti-GAD65 isoform antibodies being present in the serum or cerebrospinal fluid of 60-80% of patients with SPS [5-6]. Due to this autoimmune etiology, stiff-person syndrome is also thought to be associated with other autoimmune conditions such as diabetes mellitus type 1 [7]. SPS has also been seen in concurrence with conditions such as vitiligo, pernicious anemia, and thyroiditis [2, 8].

Diagnosing SPS should include detection of these antibodies in laboratory studies, and it must also include clinical observations and electromyographic (EMG) studies [9]. Symptomatic treatment of muscle spasms and rigidity is achieved with the use of benzodiazepines, which help to combat the uninhibited neuronal pathways. Long-term treatment of SPS includes the use of intravenous immunoglobulin, which directly addresses the autoimmune component of this disease [10]. Initially, SPS patients experience a progressive worsening of symptoms, which eventually reaches a plateau over a period that may vary from several months to several years. The combination of IVIG therapy and symptomatic management with IV lorazepam helped against the symptoms of the patients in case 1 and 2 but did not seem to control the long-term progression of disease in case 1.

## Conclusions

In this case report, we presented two cases of a rare illness: stiff-person syndrome (SPS). The first case was of an 84-year-old male with muscular spasticity in the lower extremities bilaterally. He was immobile. He tested positive for anti-GAD65 antibody. He was diagnosed with SPS and was started on intravenous immunoglobulin (IVIG), which improved his symptoms. The second case was of a 26-year-old woman, who had immune deficiency since her birth. She exhibited symptoms of neuropathy, Bell’s palsy, and muscle spasms since the age of 11. This progressed to worsening dystonia and gait disturbance, leading to complete immobility by age 16. She was also diagnosed with SPS at age 24. Both patients were treated with IVIG, irrespective of the severity of the symptoms. While the male patient did not recover fully, the female patient experienced a full recovery.

Due to the concurrence with other autoimmune disorders, patients suffering from an autoimmune condition with the aforementioned symptoms should alert the clinicians to a probable diagnosis of SPS. Despite being a rare medical condition, early recognition and treatment of SPS could probably be helpful in preventing morbidity and mortality. However, it is still premature to comment upon the importance of early recognition and treatment until future studies enhance our knowledge regarding this matter.
